# (Acetyl­acetonato-κ^2^ *O*,*O*′)[(2-bromo­phen­yl)diphenyl­phosphane-κ*P*]carbonyl­rhodium(I)

**DOI:** 10.1107/S1600536812011944

**Published:** 2012-03-24

**Authors:** Wade L. Davis, Reinout Meijboom

**Affiliations:** a Research Center for Synthesis and Catalysis, Department of Chemistry, University of Johannesburg (APK Campus), PO Box 524, Auckland Park, Johannesburg 2006, South Africa

## Abstract

In the title compound, [Rh(C_5_H_7_O_2_)(C_18_H_14_BrP)(CO)], the Rh^I^ atom adopts a slightly distorted square-planar geometry involving two O atoms [Rh—O = 2.077 (2) and 2.033 (2) Å] of the acetyl­acetonate ligand, one carbonyl C atom [Rh—C = 1.813 (2) Å] and one P atom [Rh—P = 2.242 (5) Å] of the PPh_2_(2-BrC_6_H_4_) phosphane ligand. Difference electron density maps indicate a disorder of the Br atom over two positions in an approximate 0.95:0.05 ratio. However, this disorder could not be resolved satisfactorily with the present data.

## Related literature

For background to the catalytic activity of rhodium–phosphane compounds, see: Bonati & Wilkinson (1964 ▶); Moloy & Wegman (1989 ▶); Carraz *et al.* (2000 ▶). For related rhodium structures, see: Brink *et al.* (2007 ▶); Coetzee *et al.* (2007 ▶).
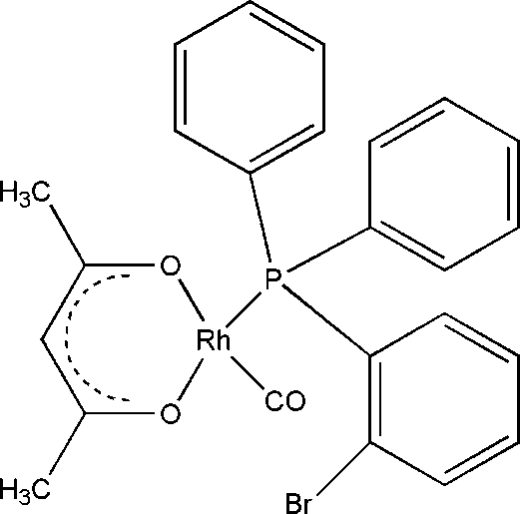


## Experimental

### 

#### Crystal data

[Rh(C_5_H_7_O_2_)(C_18_H_14_BrP)(CO)]
*M* *_r_* = 571.19
Monoclinic, 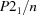

*a* = 9.0503 (2) Å
*b* = 17.8711 (4) Å
*c* = 13.9552 (3) Å
β = 102.133 (1)°
*V* = 2206.68 (8) Å^3^
*Z* = 4
Cu *K*α radiation
μ = 9.26 mm^−1^
*T* = 100 K
0.36 × 0.08 × 0.07 mm

#### Data collection

Bruker APEX DUO 4K CCD diffractometer
Absorption correction: multi-scan (*SADABS*; Bruker, 2008 ▶) *T*_min_ = 0.410, *T*_max_ = 0.753
51955 measured reflections
3830 independent reflections
3800 reflections with *I* > 2σ(*I*)
*R*_int_ = 0.040

#### Refinement

*R*[*F* ^2^ > 2σ(*F* ^2^)] = 0.019
*wR*(*F* ^2^) = 0.047
*S* = 1.14
3830 reflections
283 parameters
H-atom parameters constrained
Δρ_max_ = 0.35 e Å^−3^
Δρ_min_ = −0.47 e Å^−3^

### 

Data collection: *APEX2* (Bruker, 2010 ▶); cell refinement: *SAINT* (Bruker, 2008 ▶); data reduction: *SAINT* and *XPREP* (Bruker 2008 ▶); program(s) used to solve structure: *SIR97* (Altomare *et al.*, 1999 ▶); program(s) used to refine structure: *SHELXL97* (Sheldrick, 2008 ▶); molecular graphics: *DIAMOND* (Brandenburg & Putz, 2005 ▶); software used to prepare material for publication: *publCIF* (Westrip, 2010 ▶) and *WinGX* (Farrugia, 1999 ▶).

## Supplementary Material

Crystal structure: contains datablock(s) global, I. DOI: 10.1107/S1600536812011944/mw2054sup1.cif

Structure factors: contains datablock(s) I. DOI: 10.1107/S1600536812011944/mw2054Isup2.hkl

Additional supplementary materials:  crystallographic information; 3D view; checkCIF report

## Figures and Tables

**Fig. 1.**
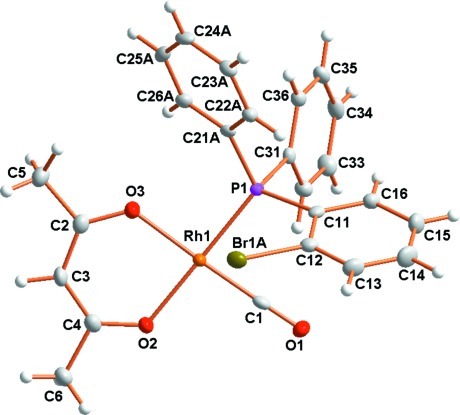
Molecular structure of the title compound, showing the atom numbering system. Displacement ellipsoids are drawn at the 50% probability level. For the C atoms in rings; the first digit indicates ring number and the second digit indicates the position of the atom in the ring. Only the highest occupancy for the disorder on the Br position is shown.

**Fig. 2.**
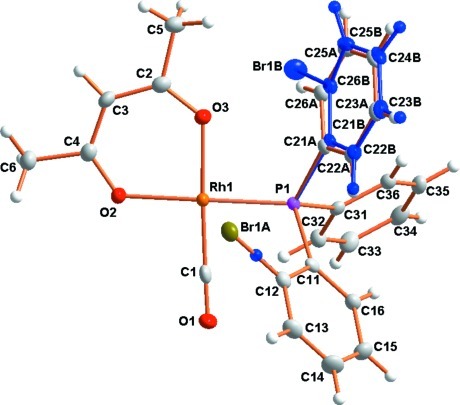
The structure of the disordered phenyl ring in (Acetylacetonato- κ2O,O')carbonyl[(2-bromophenyl)diphenyl]phosphaneκP)rhodium(I), with the minor disordered atoms with lower occupancy shown in blue.

## supplementary crystallographic information

### Comment

Acetylacetonate has two O-donor atoms with equivalent σ-electron donor capabilities. The labile carbonyl groups in dicarbonyl(acetylacetonate) rhodium(I) complexes promotes easy carbonyl displacement of one carbonyl group with a variety of phosphanes, phosphites or arsines (Bonati and Wilkinson, 1964). This work is part of an on-going investigation aimed at determining the steric effects induced by various phosphane ligands on a rhodium(I) metal centre. Previous work illustrating the catalytic importance of the rhodium(I) square- planar moieties has been conducted on rhodium mono- and di-phosphane complexes containing the symmetrical bi-dentate ligand acac (acac = acetylacetonate) (Moloy and Wegman, 1989). Symmetrical di-phosphane ligands result in the production of acetaldehyde, whereas unsymmetrical di-phosphane ligands are more stable and efficient catalysts for the carbonylation of methanol to acetic acid (Carraz *et al.*, 2000).

In the title compound, [Rh(acac)(CO){PPh_2_(2-BrC_6_H_4_)}] (acac = acetylacetonate, Ph = phenyl), the coordination around the Rh atom shows a slightly distorted square-planar arrangement, illustrated by C1—Rh1—P1 and O2—Rh1—O3 angles of 86.99 (6)° and 89.10 (6)°, respectively. The complex crystallizes in the monoclinic space group, P2(1)/n, with four molecules in the unit cell. A larger *trans*-influence of the phosphane ligand with respect to the carbonyl ligand is indicated by the longer Rh—O2 (2.077 (2) Å) bond compared to Rh—O3 (2.033 (2) Å) bond which is *trans* to the carbonyl ligand. The steric demand of the phosphane is indicated by the smaller O3—Rh1—P1 angle, (90.52 (4)°), compared to the carbonyl ligand (O2—Rh1—C1 = 93.38 (7)°). All geometric parameters are similar to previous reported complexes of the general formula [Rh(acac)(CO)*L*]; *L* = tertiary phosphane ligand (Brink *et al.* (2007); Coetzee *et al.* (2007).

We modelled the position of the Br atom as a disordered model of 95:5 occupancy over two positions. A chemically more acceptable solution is the modelling of the complete ring C21—C26 as disordered over two positions. This resulted unfortunately in an unstable refinement.

### Experimental

A solution of [Rh(acac)(CO)_2_] (42.2 mg, 0.16 mmol) in acetone (5 ml) was added slowly to a solution of [PPh_2_(2-BrC_6_H_4_)] (61.4 mg, 0.18 mmol) in acetone (5 ml). Slow evaporation of the solvent afforded the title compound as yellow crystals. Spectroscopic analysis: ^31^P{H} NMR (CDCl_3_, 162 MHz, p.p.m.): 52.4 [d, ^1^J(Rh—P) = 179.8 Hz]; IR (CH_2_Cl_2_) ν(CO): 1975.1 cm^-1^.

### Refinement

The aromatic, methine, and methyl H atoms were placed in geometrically idealized positions (C—H = 0.95–0.98) and constrained to ride on their parent atoms, with *U*_iso_(H) = 1.2*U*_eq_(C) for aromatic and methine H atoms, and *U*_iso_(H) = 1.5*U*_eq_(C) for methyl H atoms respectively. Methyl torsion angles were refined from electron density. The Br atom was modelled disorderd over two positions in a 95:5 ratio. This resulted in an unacceptably short C26—Br1B distance of 1.657 Å for the minor component.

Applying a distance restraint (SADI or *DFIX* in *SHELXL*) to the minor C26—Br1B component resulted in a severe distortion of the phenyl ring. In addition, this resulted in an unstable refinement.

Modelling the complete ring (C21—C26, Br1B) as disorderd over two positions resulted in a 96:4 ratio. This disorder provides a chemically acceptable explanation of the low occupancy of the minor disorder, as it results in distortion of the P coordination sphere. This behaviour is, however, expected in solution at room temperature. Unfortunately modelling the complete ring as a disorder resulted in an unstable refinement (results file in the supplementary information at the end of the cif file).

### Crystal data

**Table:**

[Rh(C_5_H_7_O_2_)(C_18_H_14_BrP)(CO)]	*F*(000) = 1136
*M**_r_* = 571.19	*D*_x_ = 1.719 Mg m^−^^3^
Monoclinic, *P*2_1_/*n*	Cu *K*α radiation, λ = 1.54178 Å
Hall symbol: -P 2yn	Cell parameters from 9726 reflections
*a* = 9.0503 (2) Å	θ = 4.1–65.7°
*b* = 17.8711 (4) Å	µ = 9.26 mm^−^^1^
*c* = 13.9552 (3) Å	*T* = 100 K
β = 102.133 (1)°	Needle, yellow
*V* = 2206.68 (8) Å^3^	0.36 × 0.08 × 0.07 mm
*Z* = 4	

### Data collection

**Table:**

Bruker APEX DUO 4K CCD diffractometer	3830 independent reflections
Radiation source: Incoatec IµS microfocus X-ray source	3800 reflections with *I* > 2σ(*I*)
Incoatec Quazar Multilayer Mirror monochromator	*R*_int_ = 0.040
Detector resolution: 8.4 pixels mm^-1^	θ_max_ = 66.6°, θ_min_ = 4.1°
φ and ω scans	*h* = −8→10
Absorption correction: multi-scan (*SADABS*; Bruker, 2008)	*k* = −21→21
*T*_min_ = 0.410, *T*_max_ = 0.753	*l* = −16→16
51955 measured reflections	

### Refinement

**Table:**

Refinement on *F*^2^	Primary atom site location: structure-invariant direct methods
Least-squares matrix: full	Secondary atom site location: difference Fourier map
*R*[*F*^2^ > 2σ(*F*^2^)] = 0.019	Hydrogen site location: inferred from neighbouring sites
*wR*(*F*^2^) = 0.047	H-atom parameters constrained
*S* = 1.14	*w* = 1/[σ^2^(*F*_o_^2^) + (0.0153*P*)^2^ + 2.7465*P*] where *P* = (*F*_o_^2^ + 2*F*_c_^2^)/3
3830 reflections	(Δ/σ)_max_ = 0.001
283 parameters	Δρ_max_ = 0.35 e Å^−^^3^
0 restraints	Δρ_min_ = −0.47 e Å^−^^3^

### Special details

**Table:**

Experimental. The intensity data was collected on a Bruker Apex DUO 4 K CCD diffractometer using an exposure time of 10 s/frame. A total of 3977 frames were collected with a frame width of 1.5° covering up to θ = 66.56° with 98.4% completeness accomplished.
Geometry. All e.s.d.'s (except the e.s.d. in the dihedral angle between two l.s. planes) are estimated using the full covariance matrix. The cell e.s.d.'s are taken into account individually in the estimation of e.s.d.'s in distances, angles and torsion angles; correlations between e.s.d.'s in cell parameters are only used when they are defined by crystal symmetry. An approximate (isotropic) treatment of cell e.s.d.'s is used for estimating e.s.d.'s involving l.s. planes.
Refinement. Refinement of *F*^2^ against ALL reflections. The weighted *R*-factor *wR* and goodness of fit *S* are based on *F*^2^, conventional *R*-factors *R* are based on *F*, with *F* set to zero for negative *F*^2^. The threshold expression of *F*^2^ > σ(*F*^2^) is used only for calculating *R*-factors(gt) *etc*. and is not relevant to the choice of reflections for refinement. *R*-factors based on *F*^2^ are statistically about twice as large as those based on *F*, and *R*- factors based on ALL data will be even larger.

### Fractional atomic coordinates and isotropic or equivalent isotropic displacement parameters (Å^2^)

**Table:**

	*x*	*y*	*z*	*U*_iso_*/*U*_eq_	Occ. (<1)
C1	1.0083 (2)	0.20914 (12)	0.81292 (15)	0.0170 (4)	
C2	0.7672 (3)	0.02131 (12)	0.96498 (16)	0.0202 (5)	
C3	0.7635 (3)	0.07346 (13)	1.03843 (16)	0.0227 (5)	
H3	0.7145	0.0589	1.0894	0.027*	
C4	0.8256 (3)	0.14521 (13)	1.04395 (16)	0.0199 (5)	
C5	0.7048 (3)	−0.05622 (13)	0.97335 (18)	0.0270 (5)	
H5A	0.6712	−0.0776	0.9077	0.041*	
H5B	0.619	−0.0533	1.0059	0.041*	
H5C	0.7837	−0.088	1.0118	0.041*	
C6	0.8164 (3)	0.19327 (13)	1.13130 (17)	0.0250 (5)	
H6A	0.802	0.2457	1.1107	0.038*	
H6B	0.9103	0.1884	1.1808	0.038*	
H6C	0.7309	0.177	1.1591	0.038*	
C11	0.9193 (2)	0.13799 (11)	0.60948 (15)	0.0141 (4)	
C13	0.7662 (3)	0.23761 (13)	0.51864 (17)	0.0227 (5)	
H13	0.6768	0.267	0.5069	0.027*	
C14	0.8773 (3)	0.24911 (14)	0.46495 (17)	0.0286 (5)	
H14	0.8643	0.2874	0.4166	0.034*	
C15	1.0060 (3)	0.20565 (13)	0.48098 (17)	0.0244 (5)	
H15	1.0803	0.2134	0.443	0.029*	
C16	1.0269 (3)	0.15034 (12)	0.55286 (15)	0.0178 (4)	
H16	1.1159	0.1206	0.5635	0.021*	
C21	0.8375 (2)	−0.00862 (11)	0.66851 (15)	0.0144 (4)	
C22	0.7422 (2)	−0.00895 (12)	0.57576 (15)	0.0162 (4)	
H22	0.735	0.0343	0.5354	0.019*	
C23	0.6576 (2)	−0.07239 (12)	0.54206 (16)	0.0193 (5)	
H23	0.5925	−0.0721	0.479	0.023*	
C24	0.6680 (3)	−0.13580 (12)	0.60010 (18)	0.0220 (5)	
H24	0.611	−0.1792	0.5768	0.026*	
C25	0.7624 (3)	−0.13577 (12)	0.69277 (17)	0.0208 (5)	
H25	0.7693	−0.1792	0.7327	0.025*	
C31	1.1460 (2)	0.03819 (11)	0.71559 (14)	0.0138 (4)	
C32	1.2678 (3)	0.07878 (12)	0.76843 (16)	0.0195 (5)	
H32	1.2496	0.1237	0.8005	0.023*	
C33	1.4153 (3)	0.05460 (14)	0.77495 (17)	0.0234 (5)	
H33	1.4972	0.0831	0.8108	0.028*	
C34	1.4428 (3)	−0.01132 (14)	0.72896 (16)	0.0232 (5)	
H34	1.5436	−0.0281	0.7333	0.028*	
C35	1.3233 (3)	−0.05247 (13)	0.67696 (16)	0.0207 (5)	
H35	1.3421	−0.0978	0.646	0.025*	
C36	1.1750 (2)	−0.02778 (12)	0.66963 (15)	0.0171 (4)	
H36	1.0934	−0.0561	0.6331	0.021*	
O1	1.05924 (19)	0.26162 (9)	0.78518 (12)	0.0240 (4)	
O2	0.89331 (18)	0.17500 (8)	0.98188 (11)	0.0203 (3)	
O3	0.82285 (18)	0.03145 (8)	0.88916 (11)	0.0212 (3)	
P1	0.95457 (6)	0.07227 (3)	0.71283 (4)	0.01194 (11)	
Rh1	0.923488 (17)	0.125849 (8)	0.852474 (11)	0.01343 (6)	
C12	0.7890 (2)	0.18222 (12)	0.58970 (15)	0.0168 (4)	
H12	0.7132	0.174	0.6264	0.02*	0.0419 (10)
C26	0.8466 (2)	−0.07255 (12)	0.72724 (16)	0.0177 (4)	
H26	0.9104	−0.0728	0.7907	0.021*	0.9581 (10)
Br1A	0.63052 (3)	0.167185 (13)	0.657862 (17)	0.01941 (8)	0.9581 (10)
Br1B	0.9534 (7)	−0.0921 (3)	0.8361 (4)	0.0277 (19)	0.0419 (10)

### Atomic displacement parameters (Å^2^)

**Table:**

	*U*^11^	*U*^22^	*U*^33^	*U*^12^	*U*^13^	*U*^23^
C1	0.0196 (11)	0.0181 (11)	0.0135 (10)	0.0018 (9)	0.0037 (9)	−0.0061 (8)
C2	0.0208 (12)	0.0209 (11)	0.0186 (11)	−0.0004 (9)	0.0034 (9)	0.0040 (9)
C3	0.0295 (13)	0.0242 (12)	0.0171 (11)	−0.0035 (10)	0.0112 (10)	0.0021 (9)
C4	0.0214 (12)	0.0215 (11)	0.0174 (11)	0.0032 (9)	0.0055 (9)	0.0012 (9)
C5	0.0350 (14)	0.0226 (12)	0.0241 (12)	−0.0064 (10)	0.0076 (10)	0.0027 (10)
C6	0.0302 (13)	0.0253 (12)	0.0220 (12)	0.0002 (10)	0.0111 (10)	−0.0023 (10)
C11	0.0189 (11)	0.0104 (9)	0.0115 (10)	−0.0014 (8)	−0.0003 (8)	−0.0020 (8)
C13	0.0247 (12)	0.0188 (11)	0.0217 (11)	0.0052 (9)	−0.0017 (9)	0.0025 (9)
C14	0.0395 (15)	0.0232 (12)	0.0214 (12)	0.0050 (11)	0.0024 (11)	0.0094 (10)
C15	0.0316 (13)	0.0244 (12)	0.0188 (11)	0.0014 (10)	0.0087 (10)	0.0055 (9)
C16	0.0216 (11)	0.0152 (10)	0.0166 (10)	0.0018 (9)	0.0038 (9)	0.0002 (8)
C21	0.0143 (10)	0.0131 (10)	0.0170 (10)	0.0017 (8)	0.0059 (8)	−0.0024 (8)
C22	0.0173 (11)	0.0160 (10)	0.0160 (10)	−0.0007 (8)	0.0051 (8)	−0.0016 (8)
C23	0.0174 (11)	0.0220 (11)	0.0191 (11)	−0.0029 (9)	0.0051 (9)	−0.0061 (9)
C24	0.0224 (12)	0.0162 (11)	0.0299 (13)	−0.0052 (9)	0.0114 (10)	−0.0092 (9)
C25	0.0241 (12)	0.0139 (10)	0.0271 (12)	0.0004 (9)	0.0114 (10)	0.0021 (9)
C31	0.0145 (10)	0.0161 (10)	0.0109 (9)	0.0009 (8)	0.0029 (8)	0.0036 (8)
C32	0.0207 (11)	0.0193 (11)	0.0187 (11)	−0.0001 (9)	0.0043 (9)	−0.0022 (9)
C33	0.0159 (11)	0.0323 (13)	0.0212 (11)	−0.0021 (9)	0.0019 (9)	−0.0010 (10)
C34	0.0185 (12)	0.0310 (13)	0.0217 (11)	0.0077 (10)	0.0079 (9)	0.0077 (10)
C35	0.0245 (12)	0.0204 (11)	0.0190 (11)	0.0062 (9)	0.0090 (9)	0.0017 (9)
C36	0.0192 (11)	0.0165 (10)	0.0161 (10)	0.0010 (8)	0.0050 (9)	0.0014 (8)
O1	0.0326 (9)	0.0166 (8)	0.0248 (8)	−0.0069 (7)	0.0108 (7)	−0.0023 (6)
O2	0.0281 (9)	0.0176 (8)	0.0175 (8)	−0.0026 (6)	0.0102 (7)	−0.0018 (6)
O3	0.0287 (9)	0.0183 (8)	0.0185 (8)	−0.0041 (6)	0.0093 (7)	−0.0006 (6)
P1	0.0134 (3)	0.0107 (2)	0.0116 (2)	0.00022 (19)	0.00232 (19)	−0.00053 (19)
Rh1	0.01719 (10)	0.01174 (9)	0.01218 (9)	−0.00084 (6)	0.00498 (6)	−0.00096 (5)
C12	0.0184 (11)	0.0146 (10)	0.0162 (10)	−0.0007 (8)	0.0012 (8)	−0.0034 (8)
C26	0.0177 (11)	0.0168 (11)	0.0190 (11)	0.0033 (8)	0.0052 (9)	0.0002 (8)
Br1A	0.01406 (13)	0.02069 (13)	0.02271 (14)	0.00198 (9)	0.00212 (9)	−0.00344 (9)
Br1B	0.028 (3)	0.027 (3)	0.023 (3)	0.003 (2)	−0.004 (2)	0.005 (2)

### Geometric parameters (Å, º)

**Table:**

C1—O1	1.148 (3)	C21—C26	1.398 (3)
C1—Rh1	1.813 (2)	C21—P1	1.822 (2)
C2—O3	1.277 (3)	C22—C23	1.393 (3)
C2—C3	1.391 (3)	C22—H22	0.95
C2—C5	1.510 (3)	C23—C24	1.384 (3)
C3—C4	1.395 (3)	C23—H23	0.95
C3—H3	0.95	C24—C25	1.392 (3)
C4—O2	1.278 (3)	C24—H24	0.95
C4—C6	1.508 (3)	C25—C26	1.391 (3)
C5—H5A	0.98	C25—H25	0.95
C5—H5B	0.98	C31—C36	1.393 (3)
C5—H5C	0.98	C31—C32	1.394 (3)
C6—H6A	0.98	C31—P1	1.829 (2)
C6—H6B	0.98	C32—C33	1.387 (3)
C6—H6C	0.98	C32—H32	0.95
C11—C16	1.395 (3)	C33—C34	1.389 (3)
C11—C12	1.398 (3)	C33—H33	0.95
C11—P1	1.835 (2)	C34—C35	1.381 (3)
C13—C12	1.386 (3)	C34—H34	0.95
C13—C14	1.389 (4)	C35—C36	1.396 (3)
C13—H13	0.95	C35—H35	0.95
C14—C15	1.379 (4)	C36—H36	0.95
C14—H14	0.95	O2—Rh1	2.0772 (15)
C15—C16	1.393 (3)	O3—Rh1	2.0332 (15)
C15—H15	0.95	P1—Rh1	2.2415 (5)
C16—H16	0.95	C12—H12	0.95
C21—C22	1.397 (3)	C26—H26	0.95
			
O1—C1—Rh1	177.96 (19)	C22—C23—H23	119.9
O3—C2—C3	126.2 (2)	C23—C24—C25	119.8 (2)
O3—C2—C5	114.4 (2)	C23—C24—H24	120.1
C3—C2—C5	119.4 (2)	C25—C24—H24	120.1
C2—C3—C4	125.8 (2)	C26—C25—C24	120.4 (2)
C2—C3—H3	117.1	C26—C25—H25	119.8
C4—C3—H3	117.1	C24—C25—H25	119.8
O2—C4—C3	126.2 (2)	C36—C31—C32	118.6 (2)
O2—C4—C6	115.2 (2)	C36—C31—P1	122.77 (16)
C3—C4—C6	118.6 (2)	C32—C31—P1	118.58 (16)
C2—C5—H5A	109.5	C33—C32—C31	121.0 (2)
C2—C5—H5B	109.5	C33—C32—H32	119.5
H5A—C5—H5B	109.5	C31—C32—H32	119.5
C2—C5—H5C	109.5	C32—C33—C34	119.8 (2)
H5A—C5—H5C	109.5	C32—C33—H33	120.1
H5B—C5—H5C	109.5	C34—C33—H33	120.1
C4—C6—H6A	109.5	C35—C34—C33	119.8 (2)
C4—C6—H6B	109.5	C35—C34—H34	120.1
H6A—C6—H6B	109.5	C33—C34—H34	120.1
C4—C6—H6C	109.5	C34—C35—C36	120.3 (2)
H6A—C6—H6C	109.5	C34—C35—H35	119.8
H6B—C6—H6C	109.5	C36—C35—H35	119.8
C16—C11—C12	117.34 (19)	C31—C36—C35	120.3 (2)
C16—C11—P1	121.29 (16)	C31—C36—H36	119.8
C12—C11—P1	121.15 (16)	C35—C36—H36	119.8
C12—C13—C14	118.4 (2)	C4—O2—Rh1	125.64 (14)
C12—C13—H13	120.8	C2—O3—Rh1	127.02 (14)
C14—C13—H13	120.8	C21—P1—C31	102.92 (9)
C15—C14—C13	120.8 (2)	C21—P1—C11	104.38 (9)
C15—C14—H14	119.6	C31—P1—C11	103.73 (10)
C13—C14—H14	119.6	C21—P1—Rh1	117.63 (7)
C14—C15—C16	119.9 (2)	C31—P1—Rh1	114.54 (7)
C14—C15—H15	120	C11—P1—Rh1	112.14 (7)
C16—C15—H15	120	C1—Rh1—O3	176.91 (8)
C15—C16—C11	121.0 (2)	C1—Rh1—O2	93.38 (7)
C15—C16—H16	119.5	O3—Rh1—O2	89.10 (6)
C11—C16—H16	119.5	C1—Rh1—P1	86.99 (6)
C22—C21—C26	119.26 (19)	O3—Rh1—P1	90.52 (4)
C22—C21—P1	121.32 (16)	O2—Rh1—P1	179.58 (5)
C26—C21—P1	119.39 (16)	C13—C12—C11	122.5 (2)
C23—C22—C21	120.3 (2)	C13—C12—H12	118.7
C23—C22—H22	119.8	C11—C12—H12	118.7
C21—C22—H22	119.8	C25—C26—C21	120.0 (2)
C24—C23—C22	120.2 (2)	C25—C26—H26	120
C24—C23—H23	119.9	C21—C26—H26	120
